# Therapeutic keratopigmentation Vis-à-Vis in blind eyes: complications and outcomes in a series of 184 eyes

**DOI:** 10.3389/fmed.2025.1700788

**Published:** 2025-12-10

**Authors:** Alexandre Xavier da Costa, André França Fontes Cal, Munik Pereira Bannach, Gizeli Horta de Oliveira, Giovanni Garotti

**Affiliations:** 1 Department of Ophthalmology, Federal University of São Paulo, São Paulo, Brazil; 2 Hospital de Olhos Hilton Rocha, Belo Horizonte, Brazil; 3 Hospital Santo Amaro, São Paulo, Brazil; 4 IPSEMG – Instituto de Previdência dos Servidores do Estado de Minas Gerais, Belo Horizonte, Brazil; 5 Department of Ophthalmology, Universidade de Sao Paulo, São Paulo, Brazil

**Keywords:** keratopigmentation, therapeutic keratopigmentation, keratopigmentation Vis-à-Vis, corneal tattooing, blind eye, cosmetic outcomes, corneal opacities, ocular surface surgery

## Abstract

**Purpose:**

To evaluate the outcomes, complications, and patient satisfaction following therapeutic keratopigmentation Vis-à-Vis in blind eyes.

**Methods:**

This retrospective case series included 184 eyes from blind patients who underwent KTP between 2016 and 2024, using either inorganic or mixed pigments. Superficial automated, superficial manual, and intrastromal manual approaches were performed according to corneal indication. Data collected comprised demographic information, surgical techniques, pigment type, postoperative complications, re-epithelialization time, and long-term stability. Patient-reported outcomes regarding cosmetic satisfaction and quality-of-life impact were also assessed.

**Results:**

The overall median re-epithelialization time was 14 days, though delayed healing occurred in < 20% of the patients. Postoperative complications included pigment fading, infectious keratitis, photophobia and corneal melting, though most were manageable and did not compromise final cosmetic outcomes. Cases of MRI-related ocular discomfort were noted in eyes pigmented with iron oxide–based compounds but resolved without structural alterations. At final follow-up, cosmetic appearance was graded as good to excellent in 90% of cases, and 88% of patients expressed high satisfaction, reporting improved psychosocial wellbeing.

**Conclusion:**

Therapeutic keratopigmentation Vis-à-Vis emerges as a meaningful intervention for blind eyes, not merely restoring external harmony but also restoring dignity and quality of life. Even with a tangible risk of complications, long-term pigment stability and high satisfaction rates highlight its value as both a reconstructive and humanizing procedure in corneal surgery.

## 1 Introduction

Therapeutic keratopigmentation (KTP), also known as corneal tattooing, is a surgical technique that involves the deposition of pigment within the corneal stroma to alter the eye’s appearance. It is primarily indicated in eyes with corneal opacities, iris or pupillary defects, either to improve cosmesis or to improve functional symptoms such as photophobia, diplopia, glare, or visual discomfort caused by anatomical abnormalities. Although it is typically performed in blind or severely impaired eyes, KTP can offer significant benefits in selected cases (1, 2).

Historically, corneal tattooing is among the oldest recorded ocular procedures, first mentioned by Galen and later described in greater detail by Aetius in the 5th century AD as a method to mask leucomatous opacities (3). The technique was revived in 1869 by Louis von Wecker, and since then keratopigmentation (KTP) has undergone continuous refinement. Advances in pigments, specialized needles, and dermographs—along with stricter sanitary regulations—have refined keratopigmentation into a safer and more reliable surgical option, with its progress strongly supported by the work of Alió and collaborators (1, 3). Building upon this foundation, our Vis-à-Vis approach represents the latest step in this evolution, integrating modern technology with a highly individualized, patient-centered philosophy. Beyond restoring the appearance of a damaged eye, it seeks to reestablish eye contact, social reintegration, and self-confidence—bringing the historical art of corneal tattooing into a transformative, humanistic era.

With the advent of modern micronized pigments and improved delivery techniques, KTP has become a viable therapeutic alternative for patients who are not candidates for keratoplasty or who are intolerant to cosmetic contact lenses (4–6). In such cases, KTP can restore ocular symmetry and reduce social stigma, significantly improving patients’ quality of life (7).

In many parts of the world, corneal scarring, fibrosis, and anterior segment abnormalities remain prevalent, particularly in underserved populations. In these contexts, the disfigured appearance of a blind eye may cause psychological distress and social exclusion. Studies have shown that the perception of one’s own appearance - commonly referred to as body image or self-image - plays a pivotal role in quality of life and self-esteem (8, 9). Facial disfigurements, such as a leucomatous blind eye, can lead to reduced self-confidence, negative self-image, social anxiety, fear of negative evaluation, and even social phobia (8, 9). Therefore, KTP not only serves a reconstructive function but also addresses a relevant public health issue, especially in areas with limited access to corneal transplantation or advanced ocular rehabilitation (7).

This study aims to evaluate the outcomes and complications of different therapeutic keratopigmentation techniques in blind eyes with no light perception, based on a series of cases performed by Brazilian Vis-à-Vis surgeons, providing insight into the efficacy, safety, and patient satisfaction associated with this evolving surgery.

## 2 Materials and methods

### 2.1 Study design and setting

This is a retrospective, consecutive, multicenter, non-comparative case series of patients who underwent therapeutic keratopigmentation in blind eyes with no light perception in one eye, from 2018 to 2025, performed by two Brazilian ophthalmologists. The coordinating institution for ethical oversight was the Hospital das Clínicas, Faculdade de Medicina da Universidade de São Paulo (HCFMUSP). The study was approved by the HCFMUSP Research Ethics Committee (approval number 6.690.915; CAAE 76712423.5.0000.0068) and adhered to the Declaration of Helsinki. Written informed consent was obtained from all participants or their legal guardians.

### 2.2 Participants: inclusion and exclusion criteria

A total of 182 eyes from 182 patients were included in the study. Inclusion criteria: eyes with no light perception and cosmetically disfiguring anterior segment disease (e.g., leukoma/scar, aniridia/iridodialysis, pupillary abnormalities); patients of any age; ability to comply with follow-up; signed consent. Exclusion criteria: active infectious keratitis or uncontrolled ocular surface disease at baseline; uncontrolled autoimmune disease involving the ocular surface; known allergy to pigment components; impending intraocular surgery within the early healing window; inability to suspend contact lenses or adhere to postoperative care; inability to consent. When there was recent or remote history suggestive of herpetic keratitis, surgery was deferred or performed only with peri-operative antiviral prophylaxis (see Postoperative care).

### 2.3 Data sources and variables

Charts and standardized follow-up visits (see below) were reviewed for: demographics; etiology of ocular disfigurement; surgical technique; pigment type (organic/mixed vs. inorganic mineral); re-epithelialization time; complications; follow-up length; and patient-reported outcomes (structured questionnaire on aesthetic satisfaction, discomfort, social/professional impact, and willingness to repeat the procedure).

### 2.4 Surgical techniques

Three techniques were used according to indication and surgeon preference: Superficial Automated Keratopigmentation (SAK), Superficial Manual Keratopigmentation (SMK; “Tebori de Garotti”), and Manual Intrastromal Keratopigmentation (MIK) (10, 11). Pigment colors were individually mixed chairside to match the fellow eye. All procedures were performed under asepsis with sterile instruments and sterile pigment handling.

**FIGURE 1:**
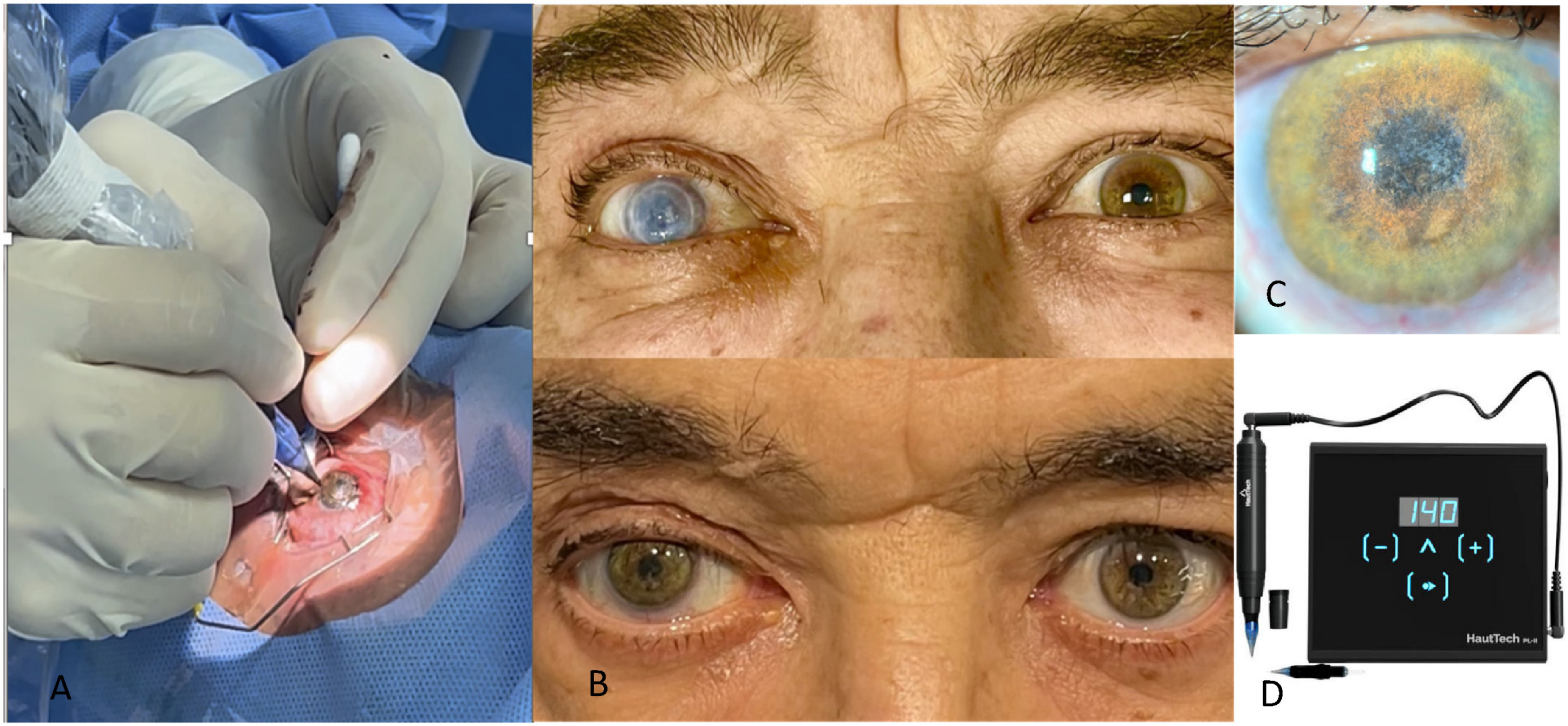
Superficial automated keratopigmentation using a dermograph. (A) Intraoperative view; (B) preoperative and 6-month postoperative photographs; (C) slit-lamp examination of the operated eye; (D) dermograph used (Precise Linear II – Haut Technology).

A. Superficial Automated Keratopigmentation (SAK) — step-by-step (Figure 1).

1. Topical anesthesia/Retrobulbar block and povidone-iodine prep; sterile draping and speculum.
2. Slit-lamp/OP microscope color check of the fellow eye; custom pigment mixture prepared immediately pre-op.
3. Ocular surface preparation with mechanical keratectomy, thermal cauterization of corneal neovascularization, and debridement/excision of superficial lesions or deposits.
4. With a dermograph puncturing device (Precise Linear II—Haut Technology) set to superficial corneal depth, multiple uniform micropunctures are created over the target zone, avoiding limbal stem cell region, using Round Liner cartridges with 1, 3 or 5 needle tips filled with pigments. Excess is irrigated with balanced salt solution.
5. Inspection for uniformity; additional passes only if needed to improve coverage;
6. Bandage contact lens (BCL) placement as indicated.

**FIGURE 2:**
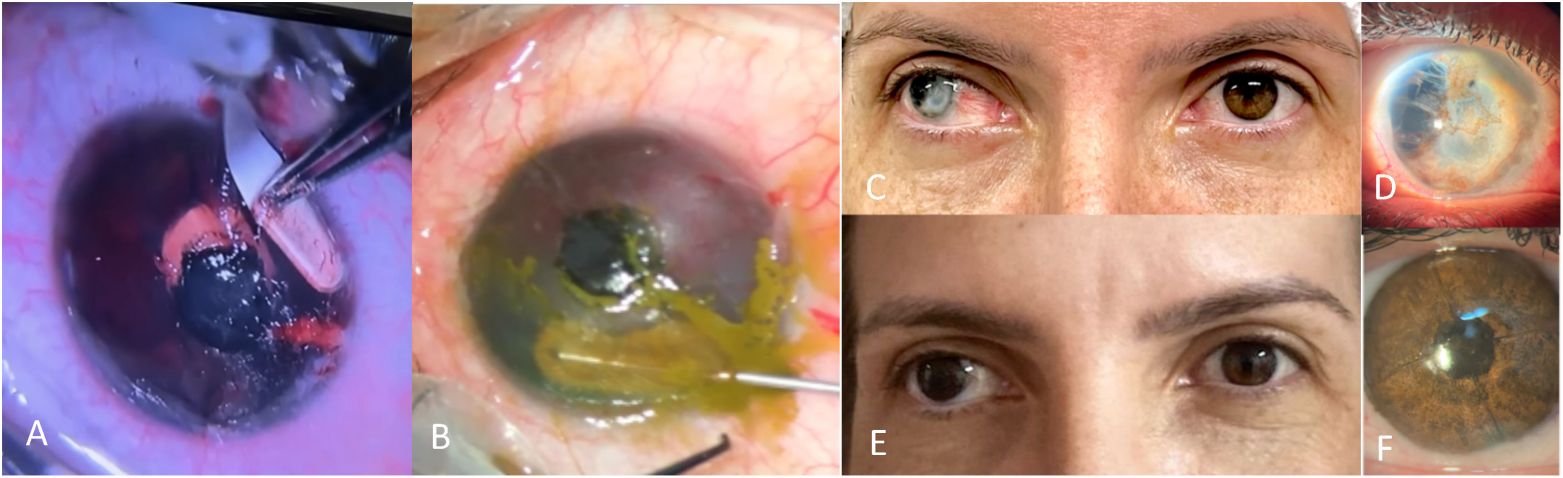
Manual intrastromal keratopigmentation technique. (A) Tunnel dissection with crescent blade; (B) intrastromal pigment implantation using a cannula; (C) preoperative photograph; (D) high-magnification view showing transparent cornea and anterior chamber disorganization; (E) 6-month postoperative photograph; (F) slit-lamp view of the healed operated eye.

B. Manual Intrastromal Keratopigmentation (MIK)—step-by-step (Figure 2).

1. Topical anesthesia/Retrobulbar block, asepsis, speculum.
2. Lamellar intrastromal pocket/tunnel creation with mini-crescent blade in a pre-planned ring or hemi-ring pattern at mid-stroma (250 μm), and a central round pocket/tunnel creation at 150 μm.
3. Cannula-assisted injection of pigment suspension into the pocket/tunnel for smooth, opaque fill; black pigment injected in the central pocket to mimic the pupil.
4. Pocket massage for even spread; irrigation of surface with balanced salt solution;
5. BCL placement as indicated.

**FIGURE 3:**
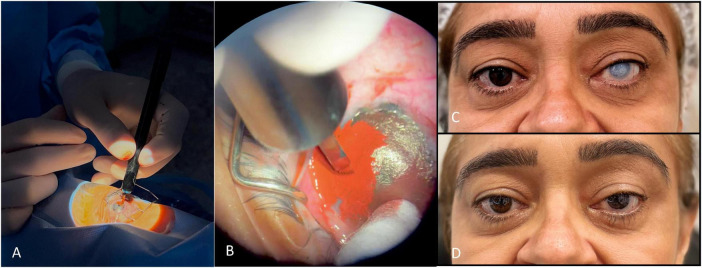
Superficial manual keratopigmentation using the “Tebori de Garotti” technique. (A) Intraoperative view; (B) close-up of pigment implantation; (C) preoperative photograph; (D) final healed result.

C. Superficial Manual Keratopigmentation (SMK; “Tebori de Garotti”)—step-by-step (Figure 3).

1. Local anesthesia and asepsis as above.
2. Ocular surface preparation with mechanical keratectomy, thermal cauterization of corneal neovascularization, and debridement/excision of superficial lesions or deposits.
3. Using a manual micro-blade/tebori instrument, controlled superficial micro-incisions are created in the anterior stroma/epithelium within the planned iris or pupil template.
4. Pigment implantation with gentle strokes to deposit material evenly within the channels.
5. Irrigation, uniformity check, and optional fine retouches for realistic iris texture (radial striae, limbal ring, pupillary shade).
6. BCL placement as indicated.

A comparative technique analysis is shown in Table 1.

**TABLE 1:** Comparative summary of techniques.

Aspect	SAK (automated)	SMK/Tebori (superficial manual)	MIK (intrastromal)
Target layer	Superficial anterior stroma	Very superficial anterior stroma	Mid-stroma pocket
Tools	Dermograph puncture device	Manual micro-blade/tebori	Crescent blade + cannula
Texture realism	Good uniformity; add shading passes	High (iris striae/limbal ring can be drawn)	High opacity/smooth fill
Typical healing	Moderate	Moderate	Faster epithelial recovery
Main risks	Epithelial defects/ulceration, fading	Epithelial defects/ulceration, fading	Microperforation (rare), pigment spread if pocket irregular

### 2.5 Pigments and preparation

**FIGURE 4:**
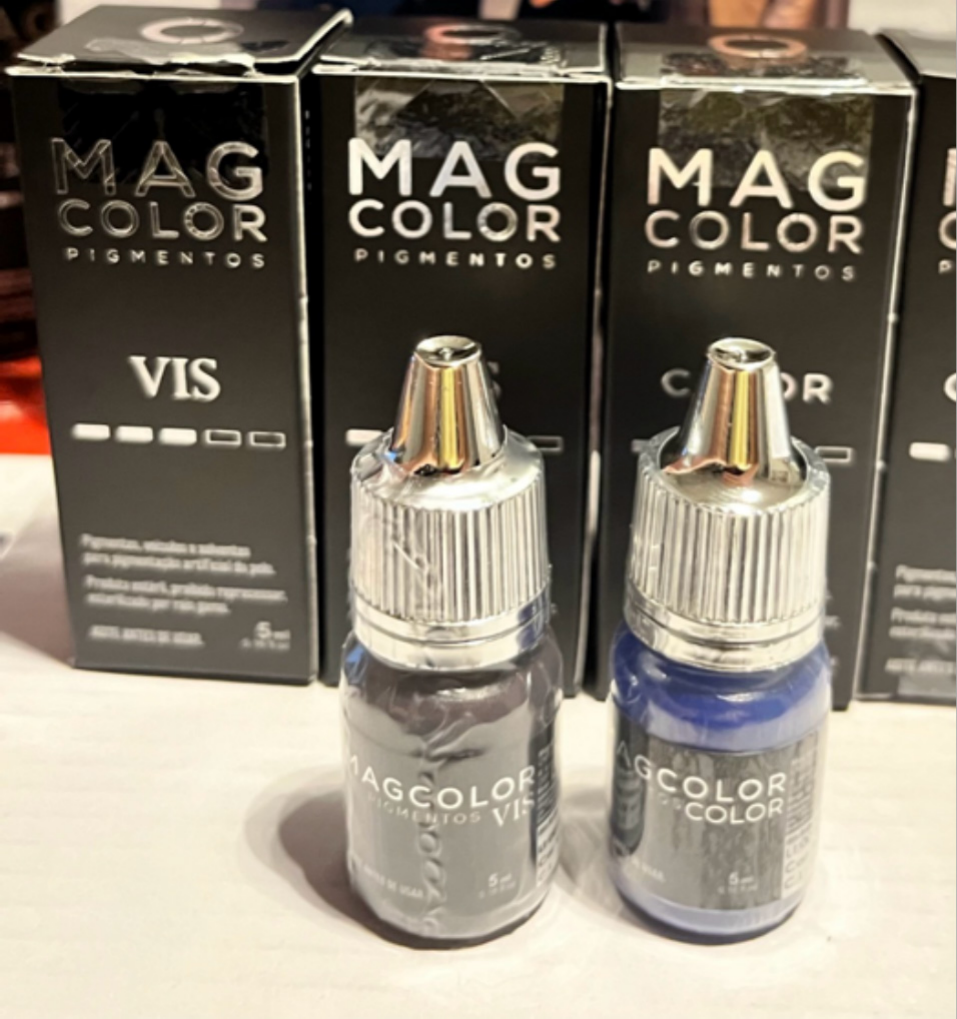
Mag color^®^ Vis pigments used for KTP.

The pigments used were products registered with the Brazilian Health Regulatory Agency (Anvisa) for dermopigmentation. Earlier cases used organic/mixed dermopigmentation inks (Electric Ink^®^), composed of deionized water, non-ionic surfactant, USP-grade vegetable glycerin, USP propylene glycol, and pigments C.I. 77266, 77891, 74160, 74260, 11741, 77891, 74160, 73915, 12475, and 21110. Later cases used micronized mineral inorganic pigments specifically adapted for KTP (Mag Color^®^ Vis) developed in collaboration with ophthalmologists, consisting of purified water, isopropyl alcohol, bidistilled glycerin, and non-toxic inorganic pigments (C.I. 77499, 77007, 77289, 77891, 77491, and 77492) (Figure 4).

In every case, pigment mixtures were prepared immediately before surgery, carefully adjusted to replicate the exact chromatic nuances of the contralateral eye. Each preparation was customized in real time, reflecting the patient’s unique iris tones and subtle variations. All handling was performed under aseptic conditions, with sterile instruments and containers, ensuring both the safety of the procedure and the preservation of pigment integrity. This individualized process embodies the essence of the Vis-à-Vis technique: tailoring the surgical act and the very material used, to achieve the most natural and human result possible.

### 2.6 Postoperative care and follow-up schedule

#### 2.6.1 Immediate regimen

Broad-spectrum topical antibiotic (e.g., fluoroquinolone) + corticosteroid qid–4 × /day for 7–14 days; Preservative-free topical lubricants hourly; protective shield at night for 1–2 weeks; strict activity and water exposure restrictions.

#### 2.6.2 Antiviral prophylaxis

Based on our series findings and literature on herpetic reactivation, we adopted routine oral acyclovir 400 mg BID for superficial KTP during the early postoperative period to reduce HSV-related complications.

#### 2.6.3 Follow-up

Day 1, week 1, then every 1–2 weeks until full re-epithelialization; thereafter at ∼1, 3, and 6 months, then yearly.

### 2.7 Outcomes

Primary outcomes: cosmetic appearance (clinical grading from photographs/slit-lamp) and patient-reported satisfaction/impact via structured questionnaire, that evaluated aesthetic satisfaction, postoperative discomfort, impact on social/professional wellbeing, and willingness to repeat the procedure (Table 2).

**TABLE 2:** Patient questionnaire.

Outcome domain	Response options
(1) Satisfaction with aesthetic outcome	1. Not satisfied 2. Satisfied 3. Very satisfied
(2) Postoperative discomfort (pain and malaise)	1. Severe 2. Moderate 3. Minimal or none
(3) Social and professional wellbeing	1. Worsened 2. Unchanged 3. Improved
(4) Would repeat the procedure	1. Yes 2. No

Secondary outcomes: time to complete re-epithelialization; pigment stability (fading/retouches); rate and type of complications; and tolerance to MRI when applicable.

### 2.8 Definition and ascertainment of complications

Complications were pre-specified and captured at each visit, with operational definitions:

1. Persistent epithelial defect (PED): non-healed epithelial loss ≥ 2 mm persisting > 14 days.
2. Infectious keratitis: stromal infiltrate with overlying epithelial defect ± anterior chamber reaction and/or hypopyon, consistent with bacterial/fungal/herpetic etiology; diagnosis based on clinical exam and therapeutic response.
3. Neurotrophic ulcer: PED/ulcer with reduced corneal sensitivity and minimal inflammation.
4. Pigment fading: visible loss of density/hue compared with baseline photographs, confirmed by masked evaluator or by surgeon consensus.
5. Microperforation: intraoperative AC breach without need for suturing or with immediate sealing.
6. Neovascularization, photophobia/persistent discomfort, phthisis bulbi: as diagnosed clinically.
7. MRI-related symptoms (if present) were documented and exams deferred until tissue stability.
8. Additional information and peculiarities about each case were individually collected and studied from the patients’ medical records.

### 2.9 Statistical analysis

Statistical analyses were performed using IBM SPSS Statistics (version 29; IBM Corp., Armonk, NY, United States). Descriptive statistics were expressed as mean ± standard deviation (SD) for continuous variables and as frequencies and percentages for categorical variables. Comparisons between groups were made using appropriate parametric or non-parametric tests according to data distribution. A *p*-value < 0.05 was considered statistically significant.

## 3 Results

Baseline characteristics are summarized in Table 3. The mean age was 44.5 years (range: 7–88), and 56% were male. Regarding the purpose of the procedure, 100% of cases were performed for therapeutic-cosmetic indications. The leading etiologies of ocular disfigurement were trauma (40%), retinal detachment (20%), congenital anomalies (11%), glaucoma (10%), uveitis (5%), and others (14%), including complicated corneal ulcers, tumors, retinal vascular occlusions and idiopathic. Regarding the technique used, SAK was used in 109 (60%) patients, MIK was used in 10 (6%), “Tebori de Garotti” SMK technique was used in 50 (28%), and combined techniques were used in 13 (6%) patients.

**TABLE 3:** Baseline demographics, clinical characteristics and techniques used.

Characteristic	Overall (*N* = 182 eyes)
Age, years	44.5 (range 7–88)
Sex	Male: 56% Female: 44%
Etiology of blindness/ disfigurement	Trauma 40%; Retinal detachment 20%; Congenital anomalies 11%; Glaucoma 10%; Uveitis 5%; Others 14%
Technique used	SAK 60%; SMK 28%; MIK 6%

The general median re-epithelialization time was 14 days, while the mean was 25.6 ± 29.1 days, reflecting the influence of a few outliers with delayed healing. Overall, 39% of patients re-epithelialized within 7 days and 58% within 15 days. Among eyes that developed postoperative ulceration (*n* = 34; 19%), the median healing time was 60 days, and the mean was 60 ± 15 days, whereas in eyes without ulceration, the median healing time was 14 days and the mean was 17 ± 14 days. When analyzing specifically the MIK cases, the mean re-epithelialization time was 7 ± 1.1 days (Table 4).

**TABLE 4:** Re-epithelialization time analysis.

Group	Median (days)	Mean ± SD (days)	Notes
General	14	25.6 ± 29.1	39% ≤ 7 days
			58% ≤ 15 days
With postoperative ulceration (*n* = 34; 19%)	60	60 ± 15	Delayed healing
Without postoperative ulceration	14	17 ± 14	Typical recovery
MIK cases (with clear cornea)	7	7 ± 1.1	Faster re-epithelialization

**FIGURE 5:**
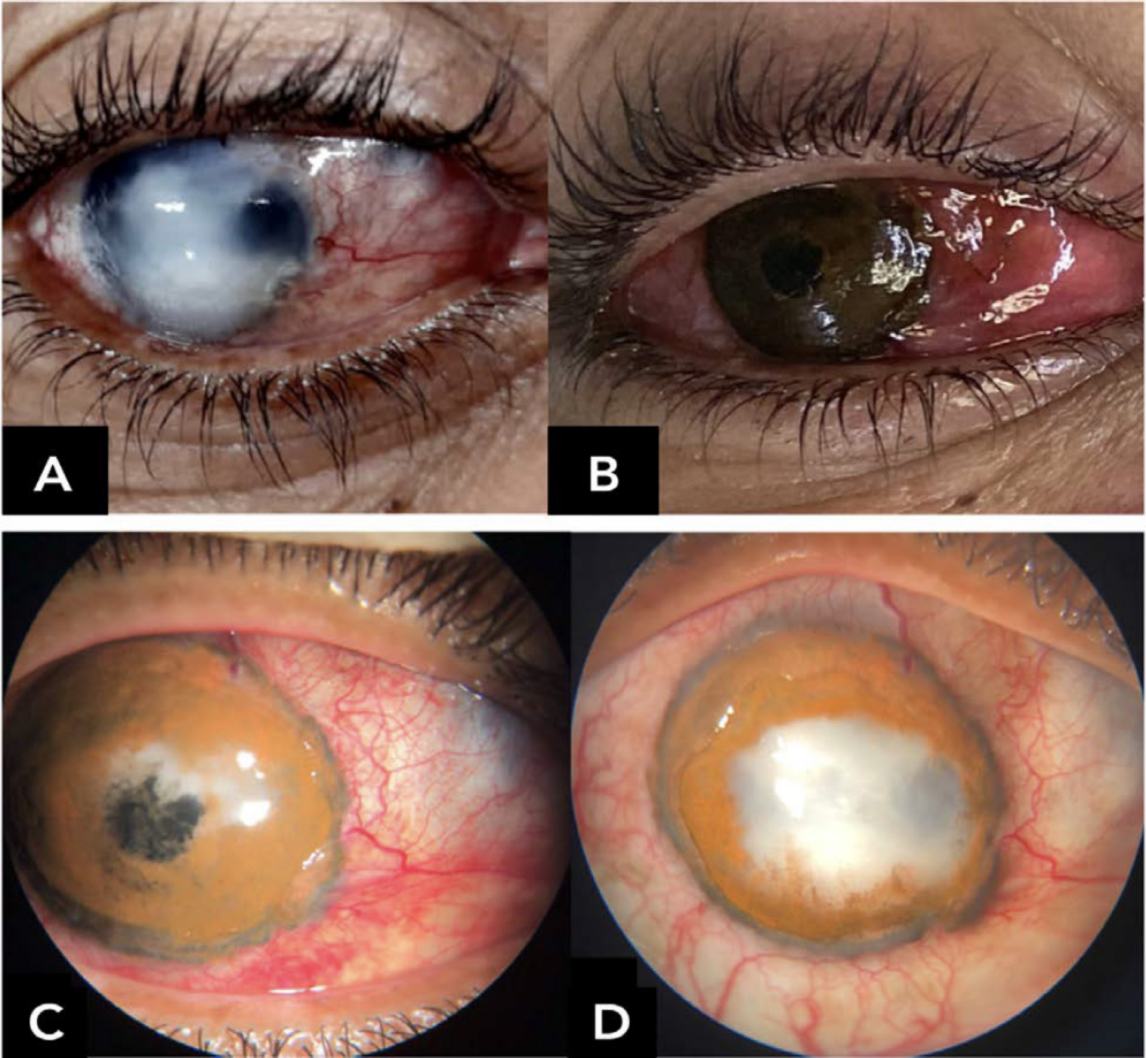
Early central corneal ulceration and melting with pigment loss within the first postoperative days, associated with corneal hypoesthesia and suggestive diagnosis of necrotizing herpetic keratitis. (A) Preoperative; (B) immediate postoperative; (C) first postoperative day; (D) postoperative day 12.

Complications were observed in 95 patients (52% of cases), the most common being pigment fading (20%) and corneal ulceration (19%), including both neurotrophic and infectious causes (Figure 5). Other complications included corneal neovascularization (3%), intraoperative microperforation (3%), and phthisis bulbi (2%; *n* = 4). Additionally, persistent ocular discomfort or photophobia were reported by 5% of patients, though these symptoms generally resolved spontaneously within 2 months postoperatively (Table 5). The median follow-up time was 180 days (6 months), with a mean of 341 ± 431 days. The longest recorded follow-up reached 6.5 years.

**TABLE 5:** Postoperative complications: definitions, frequency, timing, management, and outcomes.

Complication	Operational definition	N (eyes)	% Of eyes (*N* = 184)	Typical timing	Management (keywords)	Outcome/sequelae
Pigment fading	Visible density/hue reduction vs. baseline photos	37	20%	–	Retouching; pigment switch	Improved/stable after retouch
Persistent epithelial defect/Ulceration	Epithelial loss with/without stromal ulcer	34	19%	Early postoperative (often ≤ 14 d)	Lubrication; BCL; antivirals/antibiotics as needed	Healed; delayed re-epithelialization common
Neovascularization	New vessels in pigmented zone	6	3%	–	Topical steroids; ± anti-VEGF	Regressed/stable
Microperforation (intraoperative)	AC breach that self-sealed	6	3%	Intraoperative	Observation; BCL	No sequelae
Phthisis bulbi	Progressive ocular atrophy	4	2%	–	Supportive	Non-reversible
Photophobia/ persistent discomfort	Symptoms > 4 weeks post-op	9	5%	–	Lubricants; neuropathic pain protocol	Improved

Among the patients with a postoperative follow-up longer than 2 years (*n* = 21), seven still presented a satisfactory cosmetic result at the final evaluation. Among them, five had had an uneventful postoperative course, while two had developed infectious corneal ulcers after the surgery, that had been successfully treated. In contrast, the remaining 14 patients did not maintain a satisfactory aesthetic outcome. Four of these evolved to *phthisis bulbi*, and the other 10 had experienced significant postoperative complications, including herpetic keratitis, bacterial corneal ulcers, or corneal neovascularization. Of these, all had received organic pigments for their procedures.

Among the 23 patients who experienced postoperative pigment fading, the time to onset of depigmentation ranged from 1 to 5 years. The mean time was approximately 167 days (± 246 days), while the median time was 44 days. This wide variability reflects the heterogeneity in postoperative pigment stability, with some cases fading rapidly within days, and others only after several months or even years. Among the patients who received organic pigments (*n* = 95), 24.2% (*n* = 23) experienced postoperative pigment fading. In comparison, among those who received inorganic pigments (*n* = 71), 18.3% (*n* = 13) presented similar complications. Although the rate of depigmentation was numerically higher in the organic pigment group, the difference was not statistically significant (χ^2^ = 0.52, *p* = 0.47).

Magnetic resonance imaging (MRI)–related adverse effects were observed in one patient who underwent keratopigmentation with inorganic pigments containing iron oxide. Three months postoperatively, when attempting shoulder MRI, the patient reported severe ocular pain, hyperemia, and tearing upon merely entering the scanner room, which prevented continuation of the exam. A second attempt 1 month later caused milder discomfort, but the patient again declined to proceed. At 5 months, a third attempt allowed initiation of the exam, but fear and persistent ocular discomfort during the exam led the patient to interrupt the scan halfway, and the ocular symptoms would rapidly vanish. At that point, ophthalmologic examination revealed no abnormalities of the pigmented cornea at the slit-lamp. Six months after surgery, during a fourth attempt—this time under ophthalmologist supervision and after instillation of a topical anesthetic drop—the MRI was successfully completed. The patient reported only minimal ocular discomfort and showed mild transient hyperemia, with no evidence of corneal damage.

Among all respondents to the patient-reported outcomes questionnaire, 68% (*n* = 110) reported being “very satisfied” with the cosmetic outcome, 24% (*n* = 39) were “satisfied,” and 7% (*n* = 12) were “not satisfied.” Regarding postoperative discomfort, 55% (*n* = 89) experienced mild or no discomfort, 31% (*n* = 50) reported moderate discomfort, and 14% (*n* = 23) experienced severe discomfort in the immediate postoperative period. These symptoms were mostly self-limited. When asked about the impact of keratopigmentation on their social and professional wellbeing, 88% (*n* = 140) reported improvement, 10% (*n* = 16) perceived no change, and only 2% (*n* = 3) felt that their condition had worsened. Finally, when questioned about whether they would undergo the procedure again, 94% (*n* = 150) responded yes, while 6% (*n* = 9) said they would not.

## 4 Discussion

To the best of our knowledge, this is the largest retrospective case series of therapeutic KTP performed in blind eyes in the Americas, reinforcing the growing regional experience and relevance of this technique in the context of ocular rehabilitation. The results contribute to the growing body of literature demonstrating the safety and efficacy of KTP in managing corneal disfigurement and functional symptoms. Previous studies, such as those by Alió et al. (4, 5, 12, 13) and Hasani et al. (1), support the role of KTP as a reconstructive procedure capable of improving patient quality of life and addressing social stigma.

When analyzing the causes of blindness in the operated eyes, 60% were attributable to trauma or retinal detachment, with the majority of retinal detachment cases themselves being trauma-related and only a small proportion occurring spontaneously. Although the mean age at the time of surgery was 44 years, most patients had sustained the injury during childhood. Consequently, they had lived for decades with the visible consequences of ocular disfigurement, frequently experiencing bullying, social stigma, and the psychological burden of altered self-image.

Facial appearance plays a critical role in social interactions and self-perception, and according to Diego-Mas et al. (14), the eyes are the most influential facial features in shaping how we perceive an individual (14). Disfiguring ocular conditions can have profound psychosocial consequences, including reduced self-esteem, social anxiety, and avoidance behaviors. Studies have shown that surgical interventions aimed at restoring a more natural cosmetic appearance can improve self-confidence, reduce the impact of social stigma, and enhance overall quality of life (8, 9, 15). Within this context, therapeutic keratopigmentation offers not only a cosmetic improvement but also a pathway to psychological and social rehabilitation, enabling patients to engage more comfortably in interpersonal interactions and reintegrate more fully into their personal and professional lives (7).

The term “Vis-à-Vis” in the title of this study reflects this patient-centered vision, as the technique developed by our group prioritizes realistic, individualized outcomes, adapted to each patient’s anatomy, ocular condition, and personal expectations. Derived from the French expression meaning “face to face,” it symbolizes our goal of restoring dignity, enabling patients to meet others eye to eye, reintegrating socially, and broadening their outlook on life—both literally and figuratively.

### 4.1 Surgical techniques and healing

In this series, Superficial Automated Keratopigmentation (SAK) was the most commonly employed technique, reflecting a trend toward more standardized and reproducible procedures. Manual techniques such as SMK (Tebori de Garotti) and MIK were used in selected cases, aligned with recent innovations in pigment delivery (11, 12). Healing time varied across methods, with MIK demonstrating notably shorter re-epithelialization periods, similar to findings reported by Shymali et al. (12).

### 4.2 Pigment safety and stability

In most published studies, the pigments used for keratopigmentation are the same as those employed in dermatological tattooing or micropigmentation, consisting of a carrier base and non-toxic pigments. In Brazil, such pigments are regulated by the National Health Surveillance Agency (ANVISA) as implantable medical devices, defined as: “any device, including those that are partially or totally absorbed, intended to be fully introduced into the human body; or to replace an epithelial surface or the ocular surface, through clinical intervention, and intended to remain in that location after the intervention; or intended to be partially introduced into the human body through clinical intervention and to remain in that location after the intervention for a period of at least 30 days” (16). Pigments and their vehicles are classified as long-term surgically invasive or implantable products and fall into Risk Class III according to ANVISA regulations (17, 18). Regulatory requirements include safety assessment of all components to ensure the absence of toxic or carcinogenic substances at harmful levels, biocompatibility and chemical stability testing, and sterile manufacturing and packaging to prevent microbial contamination (16–18). In the European Union, tattoo and micropigmentation pigments must be, by definition, safe for application to skin, mucosa, and the ocular globe (19).

Tattoo pigments can be broadly categorized into organic (carbon-based) and inorganic (metal salt-based, such as iron, titanium, zinc, and chromium). Inorganic pigments tend to produce opaque, non-shiny colors, offer better stability under UV exposure, and have a lower risk of allergic reactions due to their insolubility—characteristics that make inorganic pigments the most suitable choice for keratopigmentation. Organic pigments, on the other hand, provide more vibrant colors but are more susceptible to photo-induced fading and are generally considered semipermanent. They are mainly composed of carbon, oxygen, and hydrogen, and include polycyclic and azo pigments, which can be combined with inorganic metals to expand the range of available colors (3, 20).

In our clinical practice, the initial use of organic/mixed pigments was primarily determined by product availability. The transition to inorganic pigments occurred once we identified a Brazilian manufacturer capable of producing an exclusively inorganic pigment line—Mag Color^®^ Vis—specifically adapted for keratopigmentation. This second-generation formulation featured tailored color shades and optimized viscosity, designed to better meet the surgical and aesthetic requirements of the procedure. The superior opacity, greater long-term stability, and reduced susceptibility to ultraviolet-induced fading of these inorganic pigments make them particularly suitable for achieving durable, natural-looking KTP results (3, 20).

Alió et al. (21) evaluated a keratopigmentation technique using micronized mineral pigments (Registration No. DGFPS 84-PH, Spanish Ministry of Health, 2001) composed of isopropyl alcohol 40%, water 10%, glycerin 20%, titanium dioxide C47-051 C.I. 77891 (10–30%), iron oxide C33-123 (20–30%), indigold C37-038 (15–30%), dianisidine-acetoacetanilide 20%, translucent red oxide 20%, green L-9361 20%, yellow YT-858D 20%, blue 639–4433 20%, and various black, yellow-brown, blue, and green shades in combinations designed to match the contralateral eye. Conducted in 40 eyes of 37 patients, the study reported excellent biocompatibility, no significant complications, and high cosmetic satisfaction in terms of uniformity and natural appearance. The procedure was deemed safe, effective, and minimally invasive, offering a substantial aesthetic solution for severely damaged or disfigured eyes (21).

Sirerol et al. (22) investigated the tolerance and biocompatibility of a micronized black iron oxide pigment (Registration No. DGFPS 84-PH, Spanish Ministry of Health, 2001) composed of 50% black iron oxide (C.I. 77499), yellow iron oxide (C.I. 77492), organic red pigment (C.I. 15850), ultramarine blue pigment (C.I. 77007), and 5% ethanol, propylene glycol, glycerin, drieline, amidroxy pamplemousse, and sorbitol, for use in simulated pupillary reconstruction. Through both *in vitro* and *in vivo* experiments, the pigment demonstrated excellent biocompatibility, no cytotoxicity, and minimal inflammatory response. In animal models, it showed high stability within corneal tissue without significant migration or degradation, as well as satisfactory cosmetic outcomes with uniform appearance. The authors concluded that the pigment is safe and effective for both cosmetic and functional keratopigmentation in pupillary reconstruction (22).

It is important to note that ocular siderosis—a condition caused by iron deposition in ocular tissues, usually from intraocular metallic foreign bodies releasing iron ions (23)—has not been reported in association with keratopigmentation using inorganic iron oxide pigments. These pigments are micronized, stable, and insoluble, which minimizes the release of free iron ions in ocular tissue. Available literature provides no evidence linking their use in keratopigmentation to siderosis (13, 22). Nevertheless, the procedure should be performed by qualified professionals, using high-purity materials manufactured under strict quality protocols, to ensure both safety and efficacy.

Pigment fading remains a relevant limitation of KTP. In this study, organic pigments had a numerically higher rate of postoperative fading (24.2%) compared to inorganic pigments (18.3%), although the difference was not statistically significant. Many papers have previously emphasized the improved biocompatibility and durability of micronized mineral (inorganic) pigments, corroborating our clinical observations (5, 13, 22, 24, 25).

Among patients with follow-up exceeding 2 years, only one-third maintained a satisfactory cosmetic outcome. All of these cases had received organic pigments, although existing data suggest that inorganic pigments may behave similarly over time. Unsatisfactory late outcomes were frequently associated with complications such as herpetic ulcers, corneal neovascularization or *phthisis bulbi*.

The wide range in time to depigmentation (from 1 to 5 years) underscores the variability in pigment-host interactions and the ongoing challenge of achieving long-term pigment stability. However, these findings must be considered in the context of the severe clinical conditions affecting all operated eyes, which had already undergone significant anatomical, physiological, and functional deterioration. Many were already progressing toward *phthisis* at the time of surgery, and the natural course of a blind eye is inherently unpredictable, typically involving progressive degenerative changes. Keratopigmentation does not halt this process; thus, marked changes in cosmetic appearance over time are expected, regardless of the pigment type or surgical technique used.

Still, it is important to emphasize that even patients who experienced some degree of fading years after the procedure continued to present a markedly improved aesthetic appearance compared to their preoperative condition. The vast majority remained satisfied with the results, reported a meaningful improvement in their quality of life, and stated they would undergo the procedure again.

### 4.3 MRI and magnetic pigments

Current evidence indicates that there is no mandatory waiting period after skin tattooing before undergoing MRI. Complications in tattooed patients are rare, typically limited to transient sensations of tingling or burning, without reports of permanent injury, even in tattoos containing magnetic iron oxide (26, 27). Experimental studies further demonstrated that exposure of tattooed skin to a 3T static magnetic field does not induce clinically relevant heating or cutaneous damage, regardless of pigment composition or time since tattooing (28, 29).

Nevertheless, the eye is a uniquely sensitive organ compared with the skin. The cornea has dense sensory innervation and undergoes a delicate healing process after keratopigmentation, which may predispose to heightened discomfort or intolerance during MRI. This is particularly relevant for pigments containing iron oxide, which exhibit magnetic properties and electrical conductivity, and have been implicated in MRI-related heating, discomfort, and imaging artifacts (27, 28). Thus, while the medical literature on tattoos suggests that MRI can generally be performed at any time after tattooing, our case highlights that the corneal environment may demand greater caution, with symptoms possibly related not only to pigment composition but also to the healing status of the ocular tissue.

The use of topical anesthetics in this context warrants caution. Although they can suppress pain and improve tolerance, they may also mask early signs of excessive heating, potentially increasing the risk of unrecognized corneal injury. Therefore, their use should not be routine, but considered only in selected cases under direct ophthalmologic supervision. In our patient, topical anesthesia was used after progressive improvement in tolerance with repeated MRI attempts, leading to a successful exam without structural corneal alterations. A similar case described by Alió and collaborators also reported ocular pain and hyperemia during MRI but no detectable corneal damage after exam completion (13).

In light of these observations, two practical recommendations can be made: first, the preferential use of non-magnetic inorganic pigments whenever possible, to minimize the risk of MRI-related interactions; and second, that delaying MRI examinations for approximately 6 months after keratopigmentation may be prudent, ensuring complete corneal healing and stabilization.

### 4.4 Complications

The overall complication rate in this series (52%) may seem high at first glance; however, it is consistent with the complexity and severity of the eyes included in the study. Many had a history of significant trauma, infection, or profound anatomical abnormalities, factors known to predispose to postoperative issues. The most frequent complications were pigment fading (20%) and persistent epithelial defects or ulceration (19%). These findings align with previous reports by Alió et al. (13) and Yang et al. (30), which emphasize both the potential benefits and the inherent challenges of performing corneal interventions in structurally compromised eyes. Importantly, most complications in our series were self-limiting or could be successfully managed with appropriate treatment.

The relatively high incidence of persistent epithelial defects and corneal ulcers can be explained, in large part, by preexisting ocular surface compromise. Many eyes presented with neurotrophic corneas prior to surgery, severely limiting their healing capacity. In some cases, postoperative herpetic keratitis was identified, underscoring the importance of antiviral prophylaxis. Others developed bacterial or fungal ulcers, often associated with inadequate use of prescribed topical medications or exposure to high-risk situations during the healing period, such as failure to follow rest recommendations, attending crowded environments, or engaging in activities like gardening. These observations highlight the critical importance of meticulous postoperative care, patient education, and strict adherence to follow-up, including the use of a broad-spectrum topical antibiotic and preservative free eye lubricant to minimize infectious risk and promote optimal re-epithelialization.

Prophylactic antiviral therapy with oral acyclovir was initially prescribed on a case-by-case basis, according to the clinical judgment of the attending surgeon. However, throughout the course of this case series, the emergence of herpetic keratitis in some postoperative patients—particularly those without a known prior history of herpes simplex virus—highlighted the potential for viral reactivation triggered by surgical manipulation and ocular surface inflammation (31–33). In light of these observations, our group has adopted routine antiviral prophylaxis with oral acyclovir 400 mg twice daily as a standard component of the postoperative regimen for all patients undergoing superficial keratopigmentation. This protocol aims to reduce the risk of herpetic complications, which can significantly compromise surgical outcomes and prolong healing. Notably, since the implementation of routine antiviral prophylaxis, no further cases of postoperative herpetic keratitis have been observed in our series.

### 4.5 Patient satisfaction

Despite the complication rate, patient-reported outcomes were notably favorable. Over 90% of patients expressed satisfaction with the results, 88% reported improved confidence or quality of life with considerable social/professional impact and 94% would undergo the procedure again. This aligns with data from Yilmaz et al. (7) and Urzedo et al. (34), who demonstrated the psychosocial benefits of KTP for patients with ocular disfigurement, even in the absence of visual recovery. The high levels of satisfaction reinforce the value of KTP as a patient-centered intervention.

### 4.6 Clinical implications

Therapeutic keratopigmentation should be viewed as a meaningful alternative for aesthetic and functional rehabilitation in blind eyes, particularly in settings where access to prosthetic devices or keratoplasty is limited. Its relative simplicity, outpatient feasibility, and low cost make it a pragmatic solution in both high- and low-resource environments (12, 30, 35).

### 4.7 Limitations

This study has several limitations, including its retrospective design, the absence of standardized control over pigment sources, and the lack of objective metrics for color evaluation. However, its relatively large sample size, the extended follow-up achieved in many cases, and the comprehensive analysis of complications and satisfaction provide valuable real-world evidence to guide future research and clinical decision-making. Further studies are warranted to refine keratopigmentation techniques and improve pigment formulations, with the goal of optimizing cosmetic outcomes, ensuring long-term safety, and ultimately enhancing the quality of life for patients facing the aesthetic and psychosocial challenges of disfigured, non-functional eyes.

## 5 Conclusion

Beyond achieving a natural cosmetic appearance, therapeutic keratopigmentation meaningfully improves social comfort and daily eye-to-eye interactions, marking for many the end of years of social stigma and the beginning of renewed engagement with the world. The Vis-à-Vis protocol emphasizes individualized planning (anatomy, history, and psychosocial context), careful choice among superficial and intrastromal approaches, and stable pigment formulations, yielding consistently favorable outcomes with manageable risks. These data support KTP as a practical, scalable tool in reconstructive cornea.

## Data Availability

The original contributions presented in this study are included in the article/supplementary material, further inquiries can be directed to this corresponding author.
